# Auditory and visual hallucination prevalence in Parkinson's disease and dementia with Lewy bodies: a systematic review and meta-analysis

**DOI:** 10.1017/S0033291718003161

**Published:** 2018-11-26

**Authors:** Charlotte Louise Eversfield, Llwyd David Orton

**Affiliations:** School of Healthcare Science, Manchester Metropolitan University, John Dalton Building, Chester Street, Manchester, M1 5GD, UK

**Keywords:** Auditory hallucinations, dementia with Lewy bodies, Lewy body disease, non-motor symptoms, Parkinson's disease, visual hallucinations

## Abstract

**Background:**

Non-motor features of Parkinson's disease (PD) and dementia with Lewy bodies (DLB), such as auditory hallucinations (AH), contribute to disease burden but are not well understood.

**Methods:**

Systematic review and random-effects meta-analyses of studies reporting AH associated with PD or DLB. Prevalence of visual hallucinations (VH) in identified studies meeting eligibility criteria were included in meta-analyses, facilitating comparison with AH. Synthesis of qualitative descriptions of AH was performed. PubMed, Web of Science and Scopus databases were searched for primary journal articles, written in English, published from 1970 to 2017. Studies reporting AH prevalence in PD or DLB were screened using PRISMA methods.

**Results:**

Searches identified 4542 unique studies for consideration, of which, 26 met inclusion criteria. AH pooled prevalence in PD was estimated to be 8.9% [95% confidence interval (CI) 5.3–14.5], while in DLB was estimated to be 30.8% (±23.4 to 39.3). Verbal hallucinations, perceived as originating outside the head, were the most common form of AH. Non-verbal AH were also common while musical AH were rare. VH were more prevalent, with an estimated pooled prevalence in PD of 28.2% (±19.1 to 39.5), while in DLB they were estimated to be 61.8% (±49.1 to 73.0). Meta-regression determined that the use of validated methodologies to identify hallucinations produced higher prevalence estimates.

**Conclusions:**

AH and VH present in a substantial proportion of PD and DLB cases, with VH reported more frequently in both conditions. Both AH and VH are more prevalent in DLB than PD. There is a need for standardised use of validated methods to detect and monitor hallucinations.

## Introduction

Parkinson's disease (PD) and dementia with Lewy bodies (DLB) are neurodegenerative diseases associated with *α*-synuclein dysfunction. Estimates suggest PD prevalence is 1% in people over 60 (De Lau and Breteler, 2006), while DLB has a prevalence of 0.4% in people over 65 (Vann Jones and O'Brien, 2014). Both conditions are characterised by motor dysfunction but non-motor features contribute extensively to their presentation and disease burden.

Hallucinations, spontaneous aberrant perceptions, occur in a significant proportion of cases (Diederich *et al*., 2009). Hallucinations can be induced by medications such as anticholinergics (Celesia and Wanamaker, 1972), dopamine agonists (Baker *et al*., 2009) and a range of medications modulating diverse neurochemical pathways (Porteous and Ross, 1956; Lees *et al*., 1977; Gondim *et al*., 2010; Friedman *et al*., 2011; Wand, 2012). This presents challenges to determining the causes and nature of AH in PD and DLB. The majority of hallucinations in PD and DLB are chronic, recurring and progressive in spite of stable medication regimens (Fénelon *et al*., 2000). Indeed, cognitive, sensory and circadian aspects also contribute to hallucinosis (Mosimann *et al*., 2006). Neuroleptics are often administered on presentation of AH, yet have moderate efficacy and potentially severe side effects, including increased mortality (McKeith *et al*., 1992*a*; Weintraub *et al*., 2016).

Visual hallucinations (VH) constitute a core feature of DLB diagnosis (McKeith *et al*., 2017) and have been described as a hallmark of PD (Onofrj *et al*., 2007). An associated, but distinct condition, PD dementia (PDD) (Dubois *et al*., 2007), also presents with motor and non-motor features, including hallucinations, but is under-reported in the literature. In PD, PDD and DLB, auditory hallucinations (AH) are generally considered of secondary concern, in spite of the progressive nature of AH, their contribution to loss of insight, decreased quality of life and consequent influence on the decision to move patients into long-term care (Goetz and Stebbins, 1993; Aarsland *et al*., 2000).

Previous reports vary widely in reported prevalence of AH in PD from 2% (Leu-Semenescu *et al*., 2011) to 45% (Amar *et al*., 2014; Llorca *et al*., 2016). Prevalence rates also range widely in DLB, from 18% (Suárez-González *et al*., 2014) to 43% (Piggott *et al*., 2007). Previous studies reporting prevalence of AH have predominantly been cross-sectional, with limited focus on the nature of AH reported. Furthermore, methods of determining the presence of hallucinations are diverse, potentially leading to differing reporting rates.

Aims of this study: This complex picture suggested a need to characterise, with increased precision, the prevalence and nature of AH in PD and DLB. Therefore, in this study, we aimed to conduct a systematic review and meta-analysis of studies reporting AH prevalence in PD and DLB. Furthermore, we aimed to assess the types of AH in both conditions and compared their prevalence with that of VH, which are more commonly investigated.

## Methods

Preferred Reporting Items for Systematic Reviews and Meta-Analyses (PRISMA) guidelines were used to standardise the conduct and reporting of this study. The protocol for this study was registered in advance on PROSPERO (registration number: CRD42017067337). Ethical approval for this study was awarded by the Faculty of Science and Engineering Ethics Committee at Manchester Metropolitan University (EthOS Reference Number: 0240).

### Search strategy

Literature searches for candidate studies were undertaken in the following databases: PubMed, Web of Science and Scopus. Search terms were text words: auditory, auditory hallucinations, hearing, dementia, Lewy bodies, dementia with Lewy bodies, Lewy body dementia, Parkinson's disease. The Boolean operator AND was used to maximise the number of identified papers containing combinations of search terms. A search matrix was used to ensure all paired combinations of search terms were searched for in each database.

### Study selection

The search was conducted from December 2016 to November 2017. Papers published from 1 January 1970 to 13 November 2017 were considered for inclusion. Titles and abstracts were examined to remove duplications and irrelevant studies. To be included, studies needed to (i) be written in English, (ii) report measures of AH prevalence in patients with PD or DLB, and (iii) be structured as a prospective cohort, case–control or cross-sectional study. Unpublished data were not pursued or included. Both investigators examined full-text versions of studies meeting the above criteria to assess compliance with inclusion criteria and extract data. We reviewed reference lists of all included articles to identify other potentially eligible studies.

### Data extraction and risk of bias assessment

Both authors extracted data from included papers, including study authors; publication year; study title; journal; volume; issue; pages; study design; number of participants; number of female participants; number of participants diagnosed with PD/DLB; mean age of disease onset; number of participants with AH and/or VH; qualitative descriptions of AH; time window for hallucination presentation; method of hallucination assessment. Both reviewers independently evaluated risk of bias for each study using criteria adapted from the NIH Quality Assessment Tool for Observational Cohort and Cross-Sectional Studies (https://www.nhlbi.nih.gov/health-topics/study-quality-assessment-tools). One point was awarded for each question we felt could be answered in the affirmative. Our implementation of this tool assessed study quality across nine domains: aims/objectives stated; experimental protocol appropriately described; selection bias (see Results for details); participant inclusion/exclusion criteria (selected from similar populations/appropriate diagnosis of PD or DLB); statistical analyses appropriate; condition assessed prior to outcomes; dropouts reported; timeframe for hallucination presentation sufficient; outcome measures clearly defined, valid, reliable and implemented consistently across all study participants; and presence of detailed qualitative description of AH. Scores were summed for each study to provide an overall score of bias and quality. No weighting was used in bias assessment. Studies were then grouped into those of highest (scores = 9/9), high (score = 8/9), moderate-to-high (score = 7/9), moderate (score = 6/9) and poor (score = 1–5/9) quality. Poor quality studies were excluded (Inzelberg *et al*., 1998; Katzen *et al*., 2010; Goetz *et al*., 2011; Grau-Rivera *et al*., 2013). A comparison of all binary decisions made found 77.4% agreement between the authors. Discrepancies were settled by discussion and consensus.

### Data analysis

Our primary outcome measure was AH prevalence in patients with PD or DLB. All included studies also reported prevalence of VH, which we also extracted as a secondary outcome. Where studies reported longitudinal data, we extracted the maximum values reported. Prevalence estimates in longitudinal studies were not higher than other study designs. Indeed, one longitudinal estimate (Goetz *et al*., 1998) reported the lowest prevalence of both auditory and VH in PD, suggesting this method did not bias our findings.

We conducted meta-analyses of AH and VH in Lewy body disease (LBD; pooled PD and DLB), and PD and DLB. Due to the range of study designs and different patient populations we anticipated would be included in our study, and the consequent assumption that effect sizes would be sampled from a population of effect sizes that could vary due to factors other than just sampling error, we planned to carry out random-effects models *a priori*.

We constructed random-effects models using Comprehensive Meta-Analyses software (Borenstein *et al*., 2009). We calculated pooled prevalence estimates with 95% confidence intervals (CIs) and assessed heterogeneity using the *I*^2^ statistic. Possible publication bias was assessed via the fail-safe *N*, Begg's funnel plot and Begg and Mazumdar's rank correlation tests. If publication bias was suspected, we used Duval and Tweedie's trim and fill to adjust our prevalence estimates. Outputs from these analyses were imported to an online forest plot generator to create figures (https://www.evidencepartners.com/resources/forest-plot-generator/).

Meta-regression models were created to investigate the potential contribution of study-level covariates to the observed heterogeneity in our pooled prevalence estimates. Log pooled prevalence estimate was the dependent variable, while study quality score, mean age at disease onset and the use of validated methods to detect hallucinations were set as predictive variables. Due to the diversity of methods employed to detect hallucinations, it was not possible to compare each technique. However, a clear distinction could be drawn between those studies that employed validated methods [NeuroPsychiatric Inventory (NPI), Manchester and Oxford Universities Scale for the Psychopathological Assessment of Dementia (MOUSPAD), Columbia University Scale for Psychopathology in Alzheimer's Disease (CUSPAD), Psycho-Sensory hAllucinations Scale (PSAS), University of Miami Parkinson's Disease Hallucinations Questionnaire (UM-PDHQ), Parkinson's Psychosis Rating Scale (PPRS) or Queen Square Visual Hallucination Inventory (QSVHI)] and those that did not (Rush Hallucination Inventory, semi-structured interview, questionnaire, screening hospital records or diagnostic interview and checklist).

We undertook sensitivity analyses to assess the robustness of our pooled prevalence estimate of AH and VH. We investigated the effect of year of publication by sequentially excluding studies published before 2000, 2005 and 2010. We also examined whether study design influenced outcomes by examining cross-sectional studies only and examined the effect of different quality score cut-off values for inclusion by sequentially excluding studies with scores of less than seven or eight out of nine.

## Results

### Study selection

**Fig. 1.**
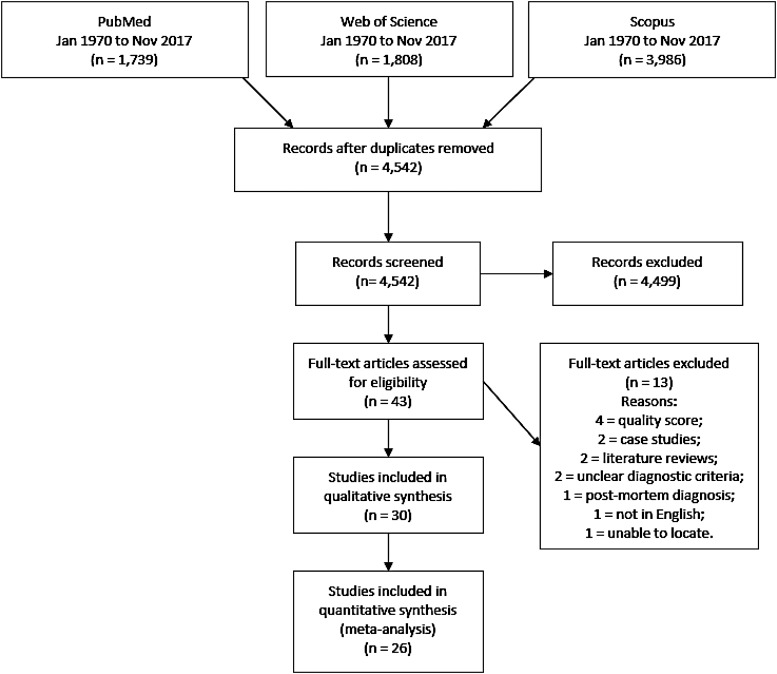
PRISMA flow diagram demonstrating study selection process.

After duplicate removal, we identified 4542 unique articles through primary database searches. Screening titles and abstracts led to the elimination of 4499 irrelevant articles. Full-text versions of the remaining 43 potentially eligible articles were assessed. Of these, 13 did not meet inclusion criteria, leaving 30 articles, published between 1992 and 2016, in the qualitative synthesis. A further four articles were excluded from quantitative meta-analyses due to low-quality assessment scores (Fig. 1). This produced 26 studies eligible for inclusion in the meta-analysis of AH prevalence (online Supplementary Table S1).

### Characteristics of included studies

Of the included studies, 19 were cross-sectional studies, four were longitudinal studies and three were case–control studies. These studies represent data from 10 countries, the majority of which were conducted in Europe (*n* = 15), while others were undertaken in North America (*n* = 6) and Asia (*n* = 5).

Five studies (Ballard *et al*., 1999; Aarsland *et al*., 2000; Ballard *et al*., 2001; Piggott *et al*., 2007; Williams *et al*., 2008) diagnosed DLB using McKeith *et al*. (1996) consensus criteria, two (Suárez-González *et al*., 2014; Shea *et al*., 2015) employed the McKeith (2006) consensus criteria, while one (Klatka *et al*., 1996) used post-mortem neuropathologic diagnosis.

Fifteen studies (Fernandez *et al*., 1992; Graham *et al*., 1997; Fénelon *et al*., 2000; Holroyd *et al*., 2001; Gupta *et al*., 2004; de Maindreville *et al*., 2005; Pacchetti *et al*., 2005; Papapetropoulos *et al*., 2008; Williams *et al*., 2008; Fénelon *et al*., 2010; Mack *et al*., 2012; Svetel *et al*., 2012; Amar *et al*., 2014; de Chazeron *et al*., 2015; Llorca *et al*., 2016) diagnosed PD according to a version of the United Kingdom Parkinson's Disease Society Brain Bank Criteria (Gibb and Lees, 1988). Five other studies used different diagnostic criteria, including definite or probable PD based on Larsen *et al*. (1994) criteria (Aarsland *et al*., 2000); Calne *et al*. (1992) criteria (Paleacu *et al*., 2005); Gelb *et al*. (1999) criteria (Lee and Weintraub, 2012); Core Assessment Program for Intracerebral Transplantations; Langston *et al*. (1992) criteria (Goetz *et al*., 1998); or criteria of Hughes *et al*. (1992) for idiopathic PD (Leu-Semenescu *et al*., 2011).

Our quality assessment rated four studies as highest quality (score = 9/9), 12 as high quality (8/9), seven as moderate-to-high quality (7/9) and four as moderate quality (6/9) (online Supplementary Table S2). As a population, study quality was weakest in reporting of qualitative descriptions of hallucinations, with other areas of quality assessment scoring being consistently high among included studies.

### Demographics of dataset

The mean age at onset of diagnosis was 61.9 years (s.d. = 7.6). We attempted to conduct group wise comparisons between diagnoses of PD without dementia (PDWD), PDD and DLB; however, PDD and PDWD were only separately reported in one study; consequently, these data were pooled into one PD group. Data from 3774 patients were identified, of which 3420 (90.6%) had PD, with the remainder DLB, and 1178 (31.2%) were female (range = 3.3–60.4%). A higher proportion of females were found in the DLB group (mean = 48.6%; range = 20.0–56.1%), than PD (mean = 30.6%; range = 3.3–60.4%). Mean age at onset for DLB was 74.1 years (s.d. = 8.1), while mean age at onset for PD was 58.8 years (s.d. = 10.6).

### Overall pooled prevalence of AH

**Fig. 2.**
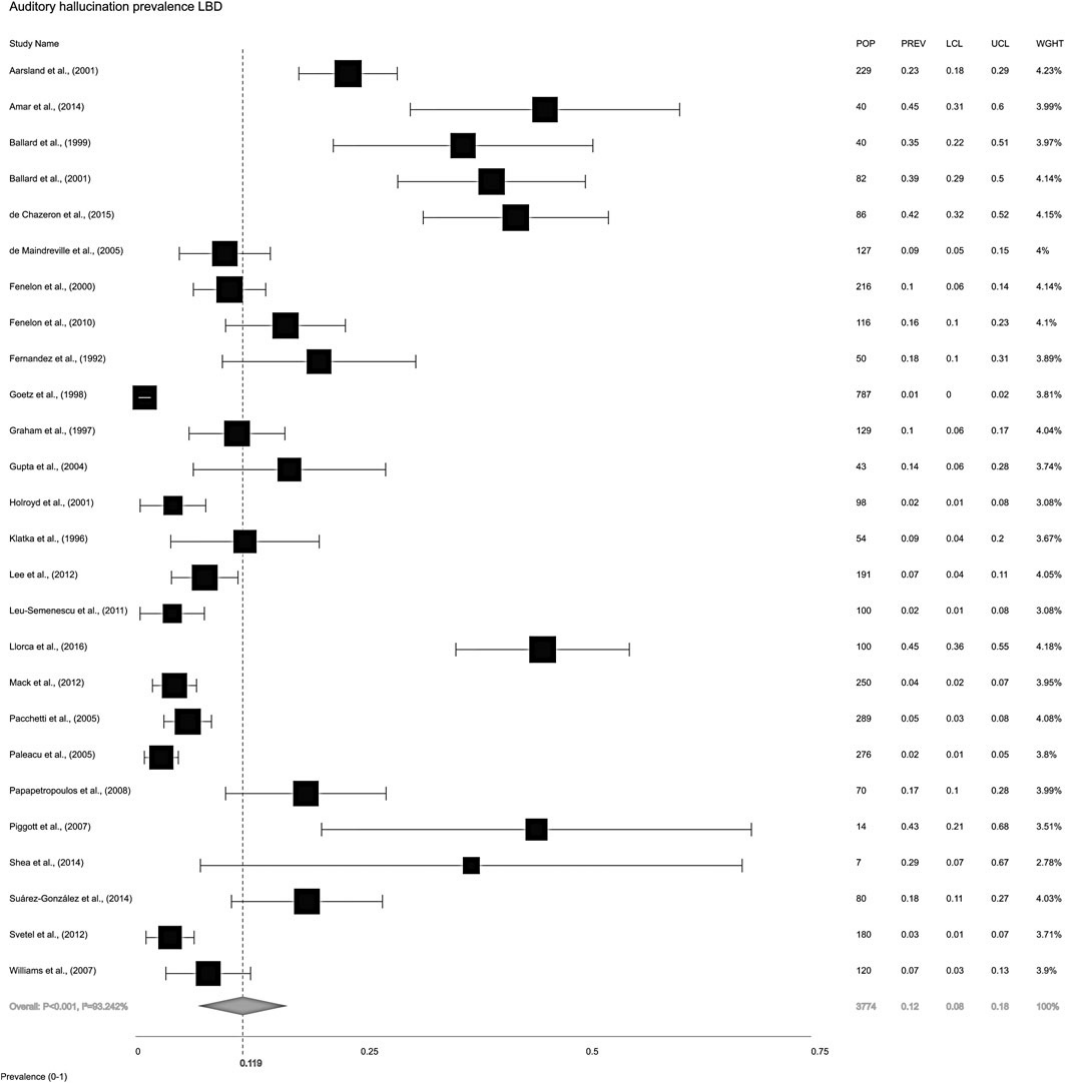
Forest plot showing random-effects model estimate of AH overall pooled prevalence of 11.9% (±7.9 to 17.7) in Lewy body disease (LBD = DLB, PDD and PDND combined). *I*^2^ = between-study heterogeneity; POP = study population; PREV = prevalence; LCL = lower confidence level; UCL = upper confidence level; WGHT = weight under random effects model.

**Fig. 3.**
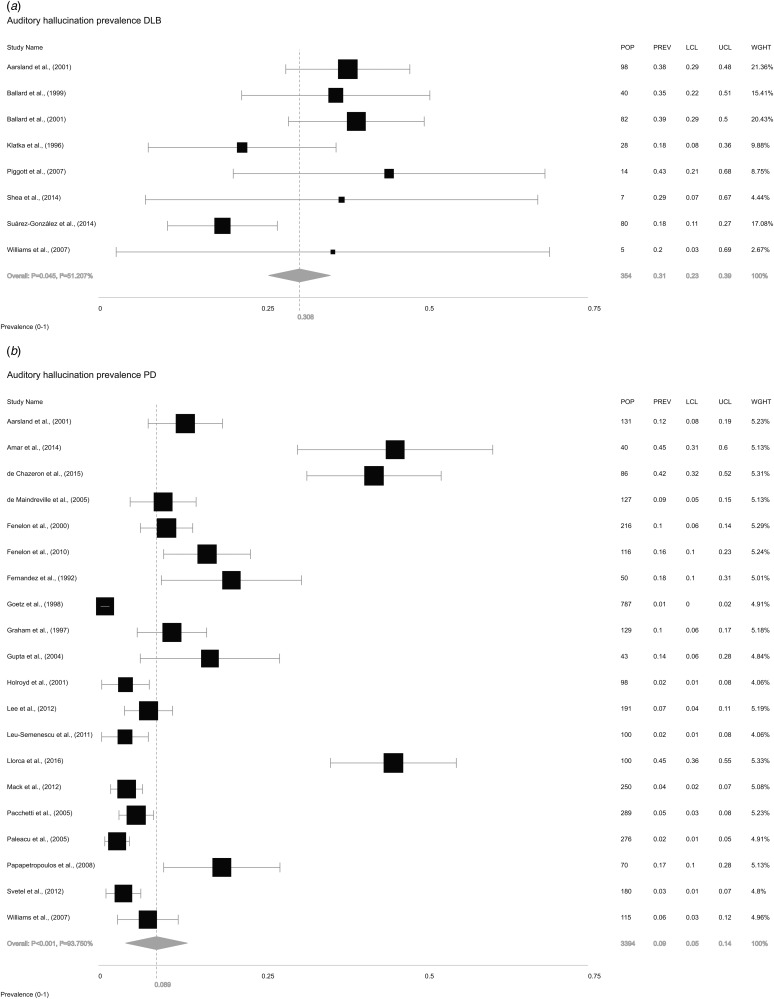
Forest plot showing random-effects model estimates of AH prevalence of 30.8% (±23.4 to 39.3) in DLB and 8.9% (±5.3 to 14.5) in PD.

The overall random-effects model pooled prevalence of AH in LBD (Fig. 2) was 11.9% (95% CI ±7.9 to 17.7). To compare the relative prevalence of AH in DLB and PD, two further random-effects models were constructed for each condition, independent of the other. Pooled prevalence of AH in DLB (Fig. 3*a*) was 30.8% (±23.4 to 39.3), while in PD (Fig. 3*b*), it was 8.9% (±5.3 to 14.5).

### Overall pooled prevalence of VH

**Fig. 4.**
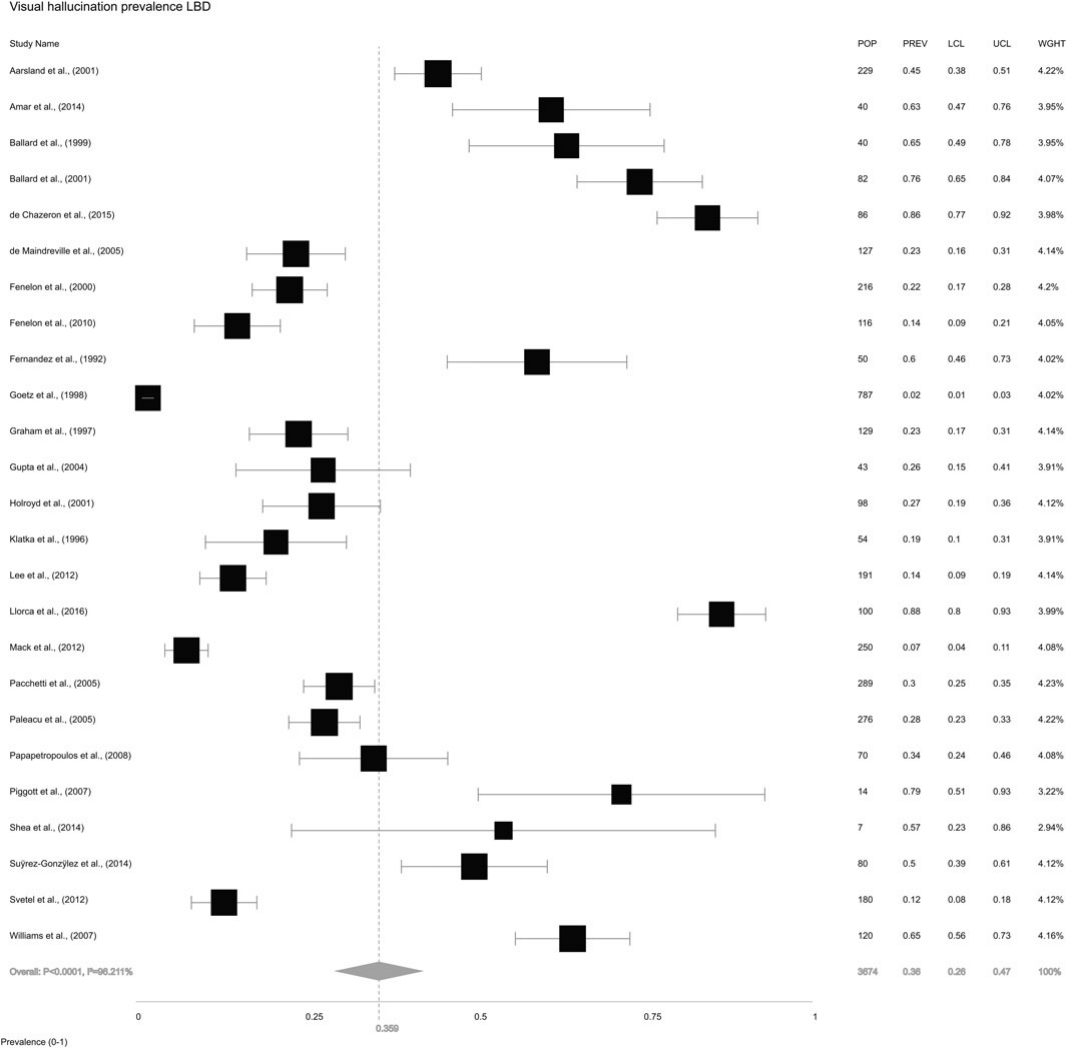
Forest plot showing random-effects model estimate of VH prevalence of 35.9% (±26.2 to 47.0) in LBD.

**Fig. 5.**
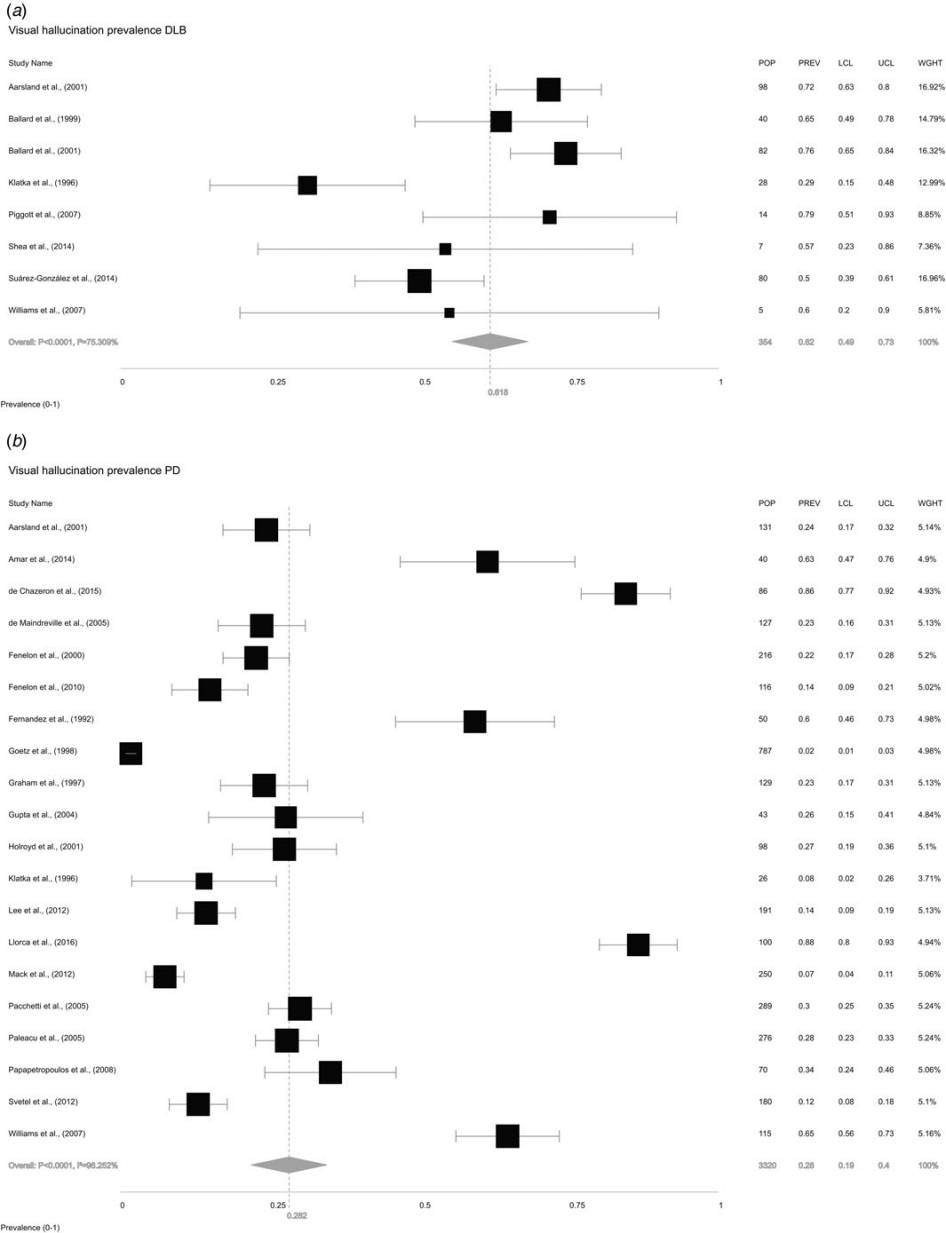
Forest plot showing random-effects model estimates of VH prevalence of 61.8% (±49.1 to 73.0) in DLB and 28.2% (±19.1 to 39.5) in PD.

We also extracted information on VH from the 26 included studies. The study by Leu-Semenescu *et al*. (2011) reported selection bias for this analysis as all 100 PD patients had VH due to the study design, and was therefore excluded leaving 25 studies in this analysis. The overall random-effects pooled prevalence of VH in LBD (Fig. 4) was 35.9% (±26.2 to 47.0). Pooled prevalence of VH in DLB (Fig. 5*a*) was 61.8% (±49.1 to 73.0), while in PD (Fig. 5*b*), it was 28.2% (±19.1 to 39.5).

### Low prevalence of single modality auditory or VH

Pure sensory hallucinations (i.e. those in only one single sensory domain) were less common. There were two (0.6%) reports of pure AH in DLB and 23 (0.7%) in PD. Pure VH were more common than auditory in both conditions, being found in 36 (10.2%) DLB cases and 122 (3.6%) PD cases. Qualitative analysis of included longitudinal studies (Graham *et al*., 1997; Goetz *et al*., 1998; Ballard *et al*., 2001; de Maindreville *et al*., 2005) revealed that pure VH tended to predate AH in both PD and DLB. As each condition progressed, AH tended to bind with recurrent complex VH to form multi-modal hallucinations that increased in prevalence from 1.5 to 10 years post baseline assessment (Goetz *et al*., 2011).

### Types of AH

The rate of reporting qualitative descriptions of AH was low, with data only available from six studies; five of which described data from PD patients, while the other described data from DLB patients.

The most commonly reported type of AH were verbal, which were reported in all six studies. Verbal AH were reported as human voices originating from outside the patient's head, often indistinct or incomprehensible, originating outside the visual field. Verbal AH were described as ‘non-threatening’, ‘non-imperative’, ‘non-congruent’ and ‘non-paranoid’. Three studies reported the proportion of AH that were verbal: Amar *et al*. (2014) found 11/18 (61.1%) of patents had verbal AH in PD; Fénelon *et al*. (2000) reported a similar proportion in PD – 13/21 (61.9%); while Suárez-González *et al*. (2014) found 5/14 (35.7%) had verbal AH in DLB.

Non-verbal sounds, such as inanimate (bullet fired, doorbell ringing, tinkling of bells, walking on steps, cracking sounds or squeaking) or animate sounds (dogs barking, tigers and lions roaring) were also common. Musical hallucinations were rare, being reported in only three patients by Fénelon *et al*. (2000), two of whom were described as ‘deaf’.

### Hallucination assessment

Across the 26 studies, there were 10 different methods employed to determine the presence of hallucinations (online Supplementary Table S1). The most common method was a semi-structured interview (*n* = 10), followed by NPI (*n* = 5), CUSPAD (*n* = 2), PSAS (*n* = 2), unnamed questionnaire (*n* = 2), UM-PDHQ (*n* = 1), MOUSPAD (*n* = 1), PPRS (*n* = 1), QSVHI (*n* = 1), screening of hospital records (*n* = 1) and not stated (=1).

### Meta-regression analyses

We observed considerable heterogeneity in all meta-analyses (*I*^2^ range = 51.2–96.3), suggesting a large proportion of the observed variance may be due to real differences between studies. To investigate whether some of the observed heterogeneity could be explained by moderator variables, such as study quality score, mean age of disease onset or method of hallucination assessment, we constructed meta-regression models for those meta-analyses comprised of sufficient study numbers (LBD and PD but not DLB). The results of these four models (online Supplementary Table S3) revealed the use of validated hallucination assessment methods could explain a significant proportion of the variance for each meta-regression (*R*^2^ range = 0.09–0.38), but study quality, mean age at disease onset and disease duration could not.

The fail-safe *N* for our meta-analysis of AH in LBD was 5649 (*Z* = −28.9; *p* < 0.0001), while there was only minor asymmetry in Begg's funnel plot (online Supplementary Fig. S6A). Begg and Mazumdar's rank correlation test suggested that publication bias was not present (Kendall's Τ-b = −0.23; *p* = 0.09) and employing Duval and Tweedie's trim and fill did not modify the random-effects effect size estimate. Similar values were found for our meta-analyses of AH in PD (online Supplementary Fig. S6B) and DLB (online Supplementary Fig. S6C), though trim and fill on the latter analysis imputed three studies that increased the pooled prevalence estimate to 37.5% (±27.7 to 48.5).

The fail-safe *N* for our meta-analysis of VHs in LBD was 1280 (*Z* = −14.16; *p* < 0.0001), while there was only minor asymmetry in Begg's funnel plot (online Supplementary Fig. S6D). Begg and Mazumdar's rank correlation test suggested that publication bias was not present (Kendall's Τ-b = −0.11; *p* = 0.44) and employing Duval and Tweedie's trim and fill did not modify the random-effects estimate. Similar values were found for our meta-analyses of VH in PD (online Supplementary Fig. S6E) and DLB (online Supplementary Fig. S6F), though trim and fill on the former analysis imputed four studies and increased the pooled prevalence estimate to 33.4% (±31.4 to 35.4).

We further investigated the potential role of cognition in hallucination status. Mini-Mental State Examination (MMSE) scores were reported in 16 studies (10 PD cases only; four DLB only; two both conditions). Pooled MMSE scores ranged from 13.2 to 29.2 (mean = 24.1). For PD, MMSE scores were lower (mean = 25.9; s.d.± = 2.3) for cases with hallucinations than those without (27.6 ± 1.2). For DLB, MMSE scores were similar for cases with hallucinations (17.3 ± 3.0) and those without hallucinations (17.2 ± 3.1). A correlation analysis found a negative relationship between MMSE scores and AH prevalence [*r*_(s)_ = −0.61; *p* = 0.008].

### Sensitivity analyses

We assessed the impact of year of study, study design and quality score on the robustness of our pooled prevalence estimates of auditory and VH in LBD. These analyses indicated that our estimates were robust, but were a few percentage points lower than analyses only including studies published from 2010 onwards or analyses only including moderate-to-high and high-quality studies (online Supplementary Table S4).

## Discussion

We report that AH and VH present in a significant proportion of PD and DLB cases, with both forms of hallucination being more prevalent in DLB. We found that VH have a higher prevalence than AH in both conditions, both occurring at rates much higher than those found in the general population (Ohayon, 2000; Waters *et al*., 2018). Of note were the wide variety of methods used to determine the presence of hallucinations. We found that more recently published studies, using validated methods, produced higher estimates of hallucination prevalence, suggesting a need for wider adoption of such approaches. Taken together, these data demonstrate that AH have a higher prevalence in PD and DLB than commonly assumed.

### Challenges to existing models of hallucinations

Existing models of recurrent complex VH have considered VH to exist in isolation from other modalities. Some models highlight dysfunctional attentional, cognitive and perceptual networks (Collerton *et al*., 2005; Diederich *et al*., 2005; Shine *et al*., 2011). Our data suggest that in PD and DLB, most cases of VH progress to become multi-modal hallucinations, incorporating a bound AH to the VH (e.g. hallucinations of people progress such that they can be heard talking). Attentional-cognitive models could account for these observations; however, sensory deficits seem incongruous with bottom-up perceptual elements of existing models. The contribution of bottom-up sensory aspects to VH has been shown via studies detailing ocular (Urwyler *et al*., 2014) and occipital lobe dysfunction (Meppelink *et al*., 2009), while central, top-down contributions involving frontal (Sanchez-Castaneda *et al*., 2010) and temporal (Harding *et al*., 2002) lobes also play a role.

However, hearing loss and auditory dysfunction are common at ages associated with PD and DLB diagnosis (Lin *et al*., 2011). It is therefore challenging to account for VH progressing to multi-modal hallucinations, binding with AH, due to a visual perceptual deficit occurring in these cases, followed by an auditory deficit. Models of simple AH, such as tinnitus, incorporate loss of peripheral drive with adaptive changes in gain (Eggermont, 1990), reductions in inhibition throughout the auditory pathway (Wang *et al*., 2011) and mismatches with central predictive coding (Sedley *et al*., 2016), yet rarely do these changes lead to more complex AH.

Attentional networks may tend to be directed more towards the visual scene, leading to more prevalent reporting of VH when these networks dysfunction. This may be due to attentional focus being more easily directed towards visual than auditory objects (Shinn-Cunningham, 2008). As widespread degeneration progresses, attentional deficits may facilitate widespread connectivity and phantom binding of VH with AH, perhaps acting via hyperexcitable cortical and subcortical networks (Grossberg, 2000; Robson *et al*., 2018).

### Strengths and limitations

Our findings are supported by the large proportion of moderate-to-high quality studies included in our meta-analyses (online Supplementary Table S2). The accuracy of our prevalence estimates are supported by the majority of studies being cross-sectional, the best experimental design by which to estimate prevalence (Mann, 2003). Furthermore, our sensitivity analyses showed that only including cross-sectional studies had little effect on our estimates (online Supplementary Table S4). However, the time window for detection of auditory or VH ranged up to 30 years post-diagnosis (Graham *et al*., 1997), limiting the temporal precision of our estimates. A selection bias may exist in our estimates due to studies selecting patients from movement disorder clinics with few community-based samples; future studies of different populations may allow insights into hallucinations in different populations.

The use of various methods to identify hallucinations was a major contributor to the high degree of heterogeneity in our meta-analyses (online Supplementary Table S3). Meta-regression models found that validated methods produced higher prevalence estimates than non-validated, suggesting that there are advantages to such approaches. However, within the range of validated measures reported in our sample (UM-PDHQ, MOUSPAD, CUSPAD, PSAS, PPRS, NPI and QSVHI) exist substantial differences in approach and outcomes. Comparisons between these approaches are beyond the scope of this study. Future work comparing these methods within the same study population may be useful. Method of hallucination assessment is an important issue, as most patients do not report AH when they first perceive them (Chou *et al*., 2005). This may be due to AH being less easily identified than visual, but could also be due to patient knowledge that AH are commonly associated with psychiatric conditions.

Our initial focus was to estimate AH prevalence in PD and DLB. As all studies included in this analysis also reported the number of participants who had VH, we also extracted and analysed these data. Numerous papers in the literature report VH but do not report other forms of hallucinations. Consequently, our estimates of VH do not contain all available evidence, but do provide comparison data that allow us to have confidence that VH are more common than AH.

Across the timespan of included studies, diagnostic criteria for PD have largely remained unchanged; while there have been multiple iterations of the consensus criteria for DLB diagnosis, each of which has modified the specificity and sensitivity of this diagnosis (Rizzo *et al*., 2017). This may be another source of between-studies heterogeneity; however, the low number of DLB studies included in our analyses did not allow meta-regression models to be constructed for these data. The lowest *I*^2^ values we observed in our meta-analyses were found for DLB studies (Figs 3*a* and 5*a*), which argues against the possible contribution of diagnostic criteria to the observed heterogeneity.

Two potentially confounding covariates were later age of diagnosis of DLB than PD and the inclusion of lower quality studies (online Supplementary Table S2). Importantly, neither of these was found to account for a significant proportion of the variance in our meta-regression models (online Supplementary Table S3). This does not exclude the possibility that age of diagnosis or age *per se* contributes to hallucinosis. Indeed, the presentation of VH in age-matched DLB and PDD patients shows extensive overlap, suggesting age-related changes may contribute to their generation (Mosimann *et al*., 2006). We were able to include data from four longitudinal cohort studies; however, two of these studies reported data for 1-year post-diagnosis, meaning that estimates of point prevalence over time were not possible in the present study. There is evidence that hallucination point prevalence in PD increases over time to affect a majority of patients (Goetz *et al*., 2005; Hely *et al*., 2008). Indeed, there is evidence that hallucinations increase in prevalence with age, post-PD diagnosis (Graham *et al*., 1997; Biglan *et al*., 2007), with VH progressing towards polysensory phenotypes (Goetz *et al*., 2011). Furthermore, once perceived, hallucinations generally recur and are accompanied by a lack of insight (Goetz *et al*., 2006), leading to increased risk of requiring placement in care facilities (Goetz and Stebbins, 1993; Aarsland *et al*., 2000).

### Types of AH

AH in LDB are complex, typically polymodal and varied in their presentation, although there is a paucity of high-quality, qualitative descriptions. Most common were verbal AH, perceived as originating outside the head, which differentiates verbal AH in PD or DLB from those found in schizophrenia. Interestingly, of the three studies to report relative rates of different types of AHs, two found that verbal hallucinations formed the majority in PD (Fénelon *et al*., 2000; Amar *et al*., 2014) while Suárez-González *et al*. (2014) reported that these were a minority in DLB. This may suggest a difference in AH presentation between the two conditions.

Non-verbal AH (acouasms) were also common and complex, whether animate or inanimate. These findings suggest auditory cortex and wider temporal and frontal lobe involvement in AH in PD and DLB, a speculation that is supported by neuroimaging data (Matsui *et al*., 2006). Some reports of simpler acouasms were also reported. The potential overlap between acouasms and tinnitus suggests that our estimated pooled prevalence of AH may be lower than the true prevalence in the population. Indeed, a recent cross-sectional study found that in a sample of 1000 patients in a cognitive neurology clinic, verbal and musical hallucinations had a prevalence of 0.9%, while tinnitus was present in 6.9% (Bayón *et al*., 2017).

AH were described as providing a soundtrack to VH (Fénelon *et al*., 2000), such as when a patient hears conversations of visually hallucinated people talking. This presentation is of note, as numerous authors described VH as preceding AH, while the polymodal combination of VH and AH may provide diagnostic utility in differentiating cognitive and functional impairment in DLB from Alzheimer's disease (Suárez-González *et al*., 2014). These data agree with early operation criteria for DLB diagnosis (McKeith *et al*., 1992*b*). While the first consensus guidelines for DLB diagnosis included AH as supportive features (McKeith *et al*., 1996), more recent updates have removed them from consideration (McKeith, 2006; McKeith *et al*., 2017). A recent analysis found that the consensus criteria for DLB had become more sensitive but less specific through these iterations, with little change in diagnostic accuracy (Rizzo *et al*., 2017). Whether the use of validated methods to detect AH is of any diagnostic utility in DLB or PD requires further investigation.

It is interesting to note that two of the three participants who reported musical hallucinations in our sample were described as ‘deaf’ (Fénelon *et al*., 2000). Musical hallucinations have been associated primarily with hearing impairment (Gordon, 1997; Cope and Baguley, 2009; Perez *et al*., 2017), though they have been reported in PD (Ergün *et al*., 2009) and DLB (Golden and Josephs, 2015) without hearing impairment. The contributions and potential interactions between hearing impairment and PD or DLB require further investigation, as there are suggestions that hearing impairment may present as a non-motor feature of PD (Lai *et al*., 2014) and hearing impairment may be more common in PD than age-matched controls (Yýlmaz *et al*., 2009).

## Conclusion

This study is the first, to our knowledge, to summarise, synthesise and contrast evidence for AH and VH prevalence in PD and DLB. AH and VH contribute to disease burden in a significant proportion of LDB cases. Methods of identification and assessment of AH and VH requires investigation to standardise measurements. Successful developments in this field may improve the accuracy of hallucination diagnosis and inform disease progression monitoring and interventions.

## Supplementary material

For supplementary material accompanying this paper visit https://doi.org/10.1017/S0033291718003161. click here to view supplementary material
