# Supplementary material for: Auditory and visual hallucination prevalence in Parkinson's disease and dementia with Lewy bodies: a systematic review and meta-analysis
Source: Psychol Med. 2018 Nov 26;49(14):2342–53. doi: 10.1017/S0033291718003161 (PMC6763539; doi:10.1017/S0033291718003161)
Supplement: Supplementary file 1 [file S0033291718003161sup001.docx]

**A**


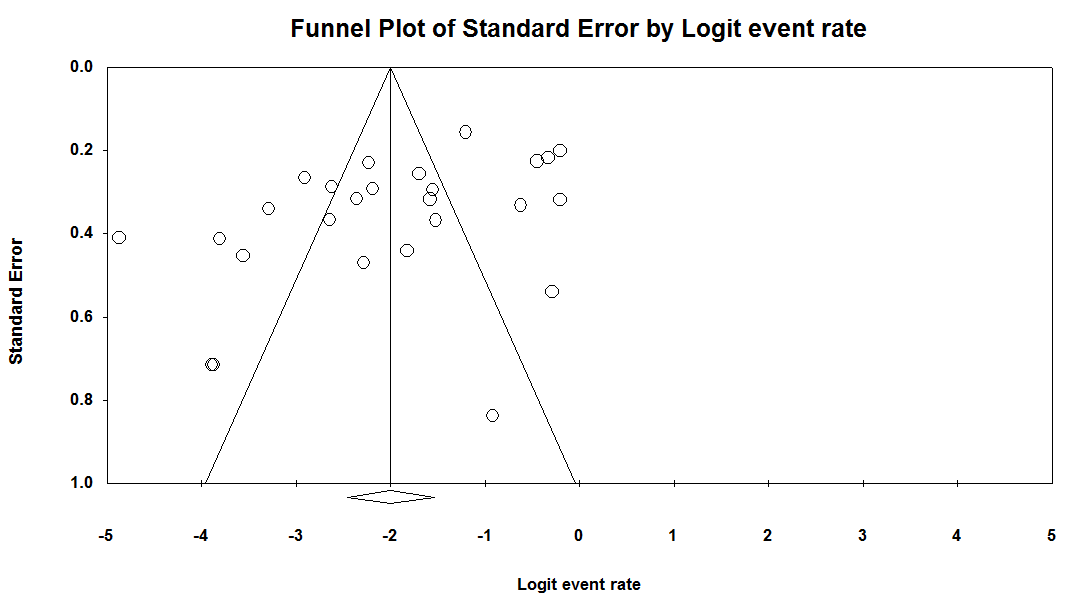


**B**


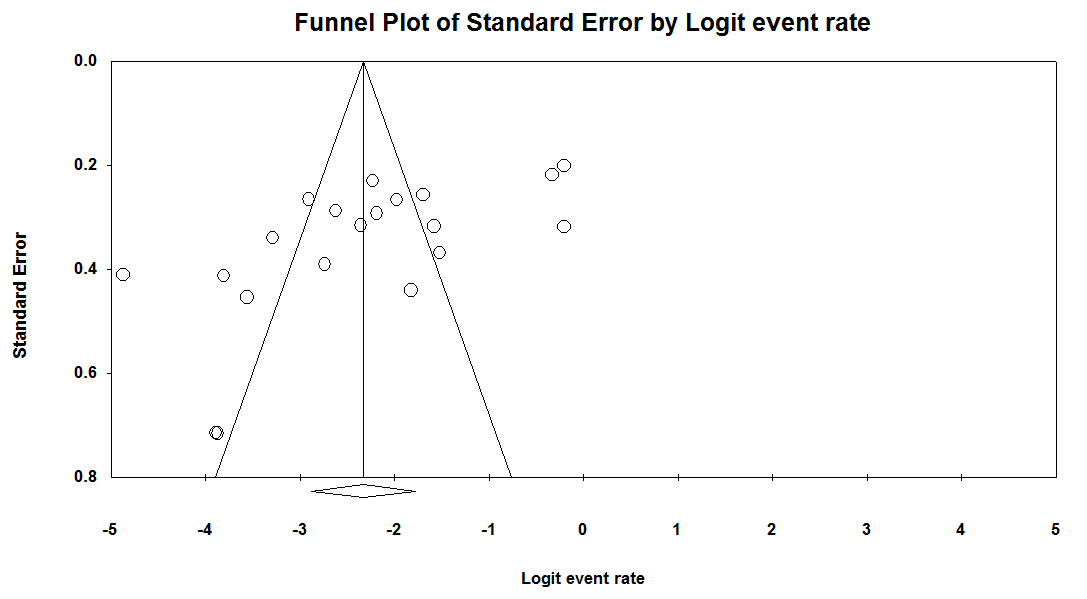


**C**


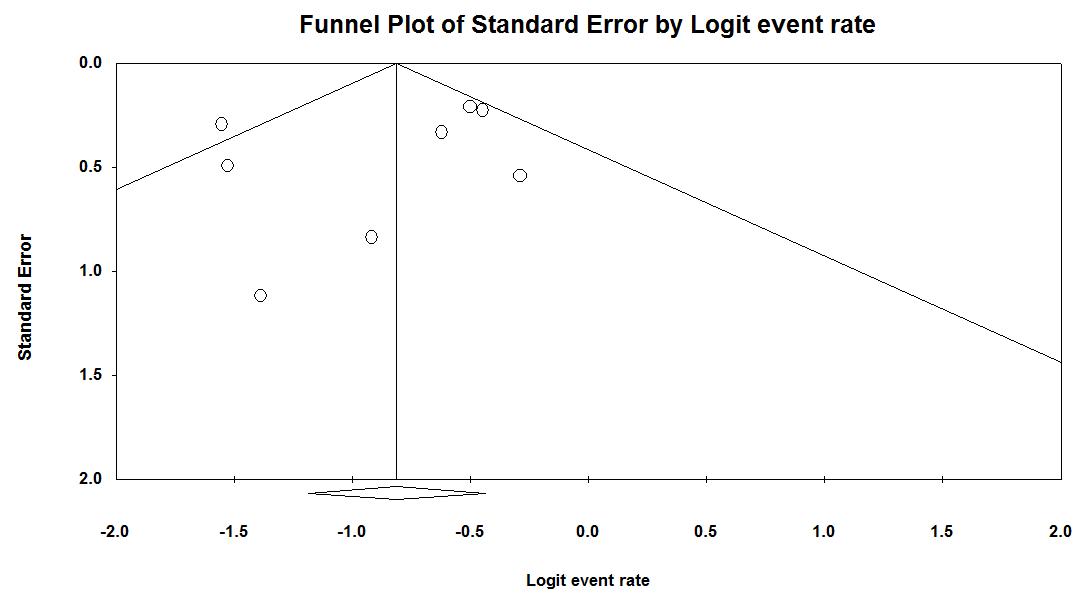


**D**


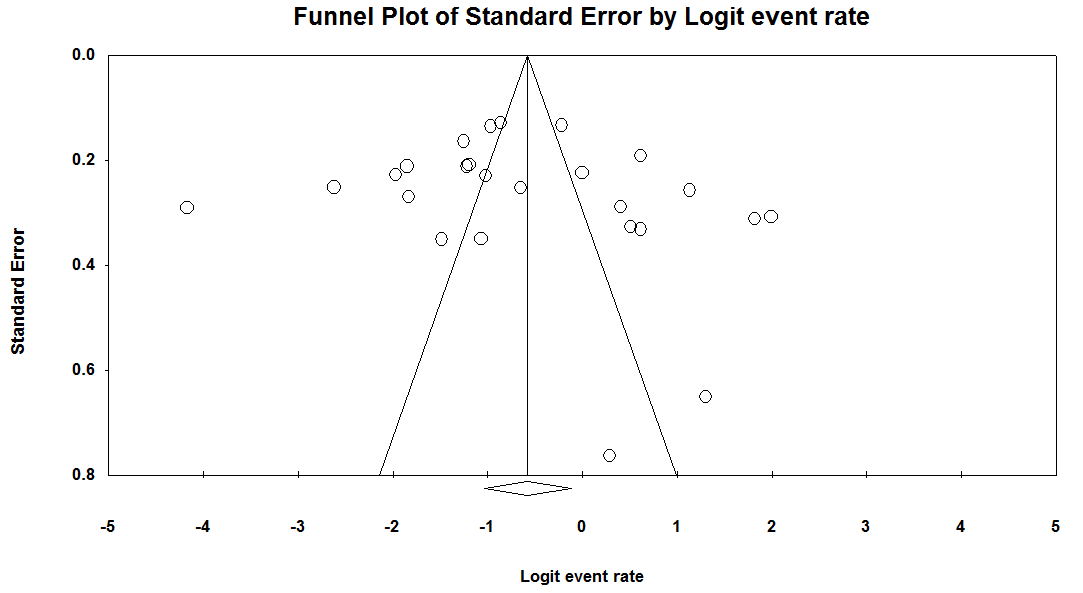


**E**


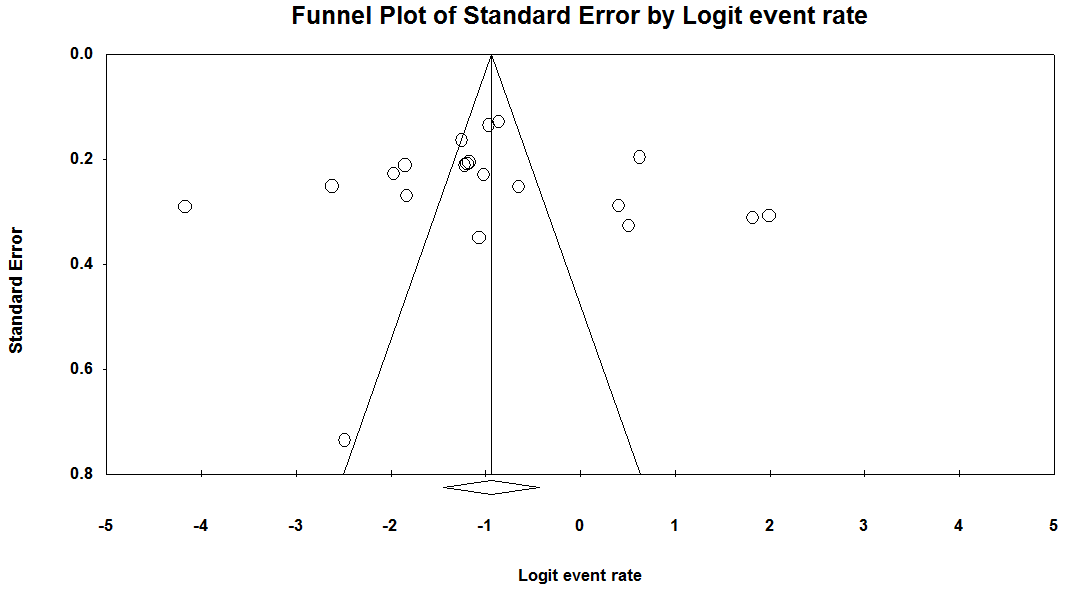


**F**


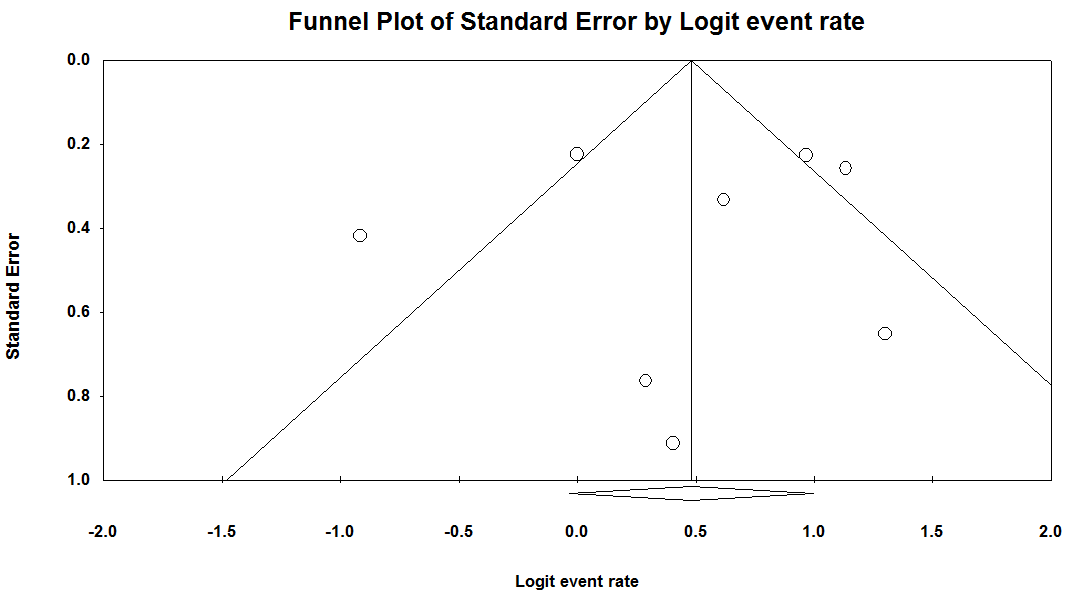


**Supplementary figure 6** Publication bias assessment by Begg’s funnel plots showing minor asymmetry with no clear evidence of publication bias in (A) auditory hallucinations in LBD, (B) PD, (C) or DLB. Similar trends were seen in (D) auditory hallucinations in LDB, (E) PD, or (F), DLB.

| **Study** | **Study design** | **# participants** | **# female** | **% female** | **Time of hallucinations presentation** | **Method of hallucination assessment** |
| --- | --- | --- | --- | --- | --- | --- |
| Aarsland et al., (2001) | Cross-sectional | 229 | 125 | 54.6 | Past month | NPI |
| Amar et al., (2014) | Cross-sectional | 40 | 5 | 12.5 | Past month | NPI |
| Ballard et al., (1999) | Cross-sectional | 270 | 154 | 57 | Past month | MOUSPAD |
| Ballard et al., (2001) | Longitudinal | 214 | 139 | 65 | Up to one year post-diagnosis | CUSPAD |
| de Chazeron et al., (2015) | Cross-sectional | 137 | 58 | 42.3 | Past 10 days | PSAS |
| de Maindreville et al., (2005) | Longitudinal | 141 | NaN | NaN | Up to one year post-diagnosis | Semi-structured interview |
| Fenelon et al., (2000) | Cross-sectional | 216 | 93 | 43.1 | Past three months | Semi-structured interview |
| Fenelon et al., (2010) | Cross-sectional | 116 | 41 | 35.3 | Unclear | Questionnaire |
| Fernandez et al., (1992) | Cross-sectional | 50 | NaN | NaN | At presentation | Not stated |
| Goetz et al., (1998) | Longitudinal | 787 | 26 | 3.3 | Within 3 months of commencement of L-DOPA | Structured interview |
| Graham et al., (1997) | Longitudinal | 129 | 60 | 46.5 | Up to 30 years | Structured interview |
| Gupta et al., (2004) | Cross-sectional | 43 | 10 | 23.3 | Unclear | Semi-structured interview |
| Holroyd et al., (2001) | Cross-sectional | 98 | 39 | 39.8 | In week prior to presentation at tertiary centre | Semi-structured interview |
| Klatka et al., (1996) | Retrospective | 112 | 64 | 57.1 | At presentation | Screening hospital records |
| Lee et al., (2012) | Cross-sectional | 191 | 54 | 28.3 | At evaluation | PPRS |
| Leu-Semenescu et al., (2011) | Cross-sectional | 100 | 34 | 34 | Any time in the past | Semi-structured interview |
| Llorca et al., (2016) | Cross-sectional | 100 | 45 | 45 | Any time in the past | PSAS |
| Mack et al., (2012) | Cross-sectional | 250 | 83 | 33.2 | At presentation | Semi-structured interview |
| Pacchetti et al., (2005) | Cross-sectional | 289 | 134 | 46.4 | Two months between first and second screening | Semi-structured interview |
| Paleacu et al., (2005) | Cross-sectional | 276 | 153 | 55.4 | Past month | Questionnaire |
| Papapetropoulos et al., (2008) | Cross-sectional | 70 | 24 | 34.3 | Active or in the past | UM-PDHQ |
| Piggott et al., (2007) | Case-control (retrospective) | 25 | 15 | 60 | Until death | Semi-structured interview |
| Shea et al., (2014) | Case-control (retrospective) | 30 | 16 | 53.3 | Past month | NPI |
| Suárez-González et al., (2014) | Cross-sectional | 165 | 91 | 55.2 | Past month | NPI & CUSPAD |
| Svetel et al., (2012) | Cross-sectional | 180 | 85 | 47.2 | Past three months | NPI |
| Williams et al., (2007) | Cross-sectional | 181 | 78 | 43.1 | Unclear | QSVHI |

**Supplementary table 1** Overview of study characteristics included in meta-analyses.

| **Study** | **Aims stated** | **Experimental protocol** | **Baseline criteria** | **Inclusion and exclusion criteria** | **Statistical analyses** | **Outcome assessment** | **Timeframe for hallucinations** | **Outcome measures** | **Description of hallucinations** | **Total** |
| --- | --- | --- | --- | --- | --- | --- | --- | --- | --- | --- |
| Aarsland et al., (2001) | 1 | 1 | 1 | 1 | 1 | 1 | 1 | 1 | 0 | 8 |
| Amar et al., (2014) | 1 | 1 | 1 | 1 | 1 | 1 | 1 | 1 | 1 | 9 |
| Ballard et al., (1999) | 1 | 1 | 1 | 1 | 1 | 1 | 1 | 1 | 0 | 8 |
| Ballard et al., (2001) | 1 | 0 | 1 | 1 | 1 | 1 | 1 | 1 | 0 | 7 |
| de Chazeron et al., (2015) | 1 | 1 | 1 | 1 | 1 | 1 | 1 | 1 | 0 | 8 |
| de Maindreville et al., (2005) | 1 | 1 | 0 | 1 | 1 | 1 | 1 | 1 | 0 | 7 |
| Fenelon et al., (2000) | 1 | 1 | 1 | 1 | 1 | 1 | 0 | 1 | 0 | 7 |
| Fenelon et al., (2010) | 1 | 1 | 1 | 1 | 1 | 1 | 1 | 1 | 1 | 9 |
| Fernandez et al., (1992) | 0 | 1 | 0 | 1 | 1 | 1 | 1 | 1 | 0 | 6 |
| Goetz et al., (1998) | 1 | 1 | 0 | 1 | 0 | 1 | 1 | 1 | 0 | 6 |
| Graham et al., (1997) | 1 | 1 | 1 | 1 | 1 | 1 | 0 | 1 | 1 | 8 |
| Gupta et al., (2004) | 1 | 1 | 1 | 1 | 0 | 1 | 0 | 1 | 1 | 7 |
| Holroyd et al., (2001) | 1 | 1 | 0 | 1 | 1 | 1 | 1 | 1 | 0 | 7 |
| Klatka et al., (1996) | 1 | 1 | 1 | 1 | 0 | 1 | 1 | 0 | 0 | 6 |
| Lee et al., (2012) | 1 | 1 | 1 | 1 | 1 | 1 | 1 | 0 | 0 | 7 |
| Leu-Semenescu et al., (2011) | 1 | 1 | 1 | 1 | 1 | 1 | 1 | 0 | 1 | 8 |
| Llorca et al., (2016) | 1 | 1 | 1 | 1 | 1 | 1 | 1 | 1 | 0 | 8 |
| Mack et al., (2012) | 1 | 1 | 1 | 1 | 1 | 1 | 1 | 1 | 0 | 8 |
| Pacchetti et al., (2005) | 1 | 1 | 1 | 1 | 1 | 1 | 1 | 1 | 0 | 8 |
| Paleacu et al., (2005) | 1 | 1 | 1 | 1 | 1 | 1 | 1 | 1 | 0 | 8 |
| Papapetropoulos et al., (2008) | 1 | 1 | 1 | 1 | 1 | 1 | 1 | 1 | 0 | 8 |
| Piggott et al., (2007) | 1 | 1 | 1 | 1 | 1 | 1 | 1 | 1 | 0 | 8 |
| Shea et al., (2014) | 1 | 1 | 0 | 1 | 1 | 1 | 1 | 1 | 0 | 7 |
| Suárez-González et al., (2014) | 1 | 1 | 1 | 1 | 1 | 1 | 1 | 1 | 1 | 9 |
| Svetel et al., (2012) | 1 | 1 | 1 | 1 | 1 | 1 | 1 | 1 | 0 | 8 |
| Williams et al., (2007) | 1 | 1 | 1 | 1 | 0 | 1 | 0 | 1 | 0 | 6 |

**Supplementary table 2** Quality assessment of studies included in quantitative synthesis.

| **Auditory hallucinations in LBD Meta-regression model, random effects, Z-distribution, Logit event rate (R^2^=0.38)** | | | | | | |
| --- | --- | --- | --- | --- | --- | --- |
| **Covariate** | **Coefficient** | **Standard error** | **LCL** | **UCL** | **Z-value** | **2-sided p-value** |
| Intercept | -4.61 | 2.3 | -9.11 | -0.11 | -2.01 | 0.05 |
| Study quality | 0.19 | 0.21 | -0.23 | 0.6 | 0.86 | 0.39 |
| Mean age at disease onset | 0.01 | 0.03 | -0.04 | 0.06 | 0.33 | 0.74 |
| Hallucinations assessed by validated methods yes/no | 1.34 | 0.41 | 0.53 | 2.15 | 3.24 | 0.001 |
| **Auditory hallucinations in PD Meta-regression model, random effects, Z-distribution, Logit event rate (R^2^=0.27)** | | | | | | |
| **Covariate** | **Coefficient** | **Standard error** | **LCL** | **UCL** | **Z-value** | **2-sided p-value** |
| Intercept | -3.51 | 2.51 | -8.42 | 1.4 | -1.4 | 0.161 |
| Study quality | 0.18 | 0.31 | -0.43 | 0.79 | 0.59 | 0.56 |
| Mean age at disease onset | -0.01 | 0.02 | -0.04 | 0.02 | -0.71 | 0.48 |
| Hallucinations assessed by validated methods yes/no | 1.22 | 0.5 | 0.23 | 2.21 | 2.42 | 0.015 |
| **Visual hallucinations in LBD Meta-regression model, random effects, Z-distribution, Logit event rate (R^2^=0.18)** | | | | | | |
| **Covariate** | **Coefficient** | **Standard error** | **LCL** | **UCL** | **Z-value** | **2-sided p-value** |
| Intercept | -2.59 | 2.53 | -7.54 | 2.36 | -1.03 | 0.3 |
| Study quality | 0.02 | 0.24 | -0.45 | 0.49 | 0.08 | 0.94 |
| Mean age at disease onset | 0.02 | 0.03 | -0.04 | 0.07 | 0.59 | 0.55 |
| Hallucinations assessed by validated methods yes/no | 1.54 | 0.46 | 0.64 | 2.45 | 3.36 | 0.0008 |
| **Visual hallucinations in PD Meta-regression model, random effects, Z-distribution, Logit event rate (R^2^=0.09)** | | | | | | |
| **Covariate** | **Coefficient** | **Standard error** | **LCL** | **UCL** | **Z-value** | **2-sided p-value** |
| Intercept | -1.54 | 2.37 | -6.18 | 3.1 | -0.65 | 0.52 |
| Study quality | 0.16 | 0.3 | -0.43 | 0.74 | 0.53 | 0.6 |
| Mean age at disease onset | -0.02 | 0.02 | -0.05 | 0.02 | -1.05 | 0.29 |
| Hallucinations assessed by validated methods yes/no | 1.38 | 0.55 | 0.3 | 2.47 | 2.51 | 0.01 |

**Supplementary table 3** Output from four meta-regression models. All models found that the use of validated methods to detect hallucinations produced higher estimated of prevalence and could account for a significant proportion of the variance. Neither study quality or mean age of disease onset were found to account for a significant proportion of the observed heterogeneity.

| **Sensitivity analyses auditory hallucinations in LDB** | | |  |  |  |  |
| --- | --- | --- | --- | --- | --- | --- |
| **Analyses** | **# studies** | **# participants** | **prevalence (%)** | **LCL 95%** | **ULD 95%** | **I^2^ (%)** |
| 2000 and later studies only | 21 | 2714 | 12.7 | 8.1 | 19.2 | 93.1 |
| 2005 and later studies only | 16 | 2046 | 12.2 | 6.9 | 20.8 | 93.8 |
| 2010 and later studies only | 12 | 1291 | 15.2 | 8 | 27.1 | 93.6 |
| Cross-sectional studies only | 20 | 2628 | 11.7 | 7.5 | 17.8 | 92.8 |
| Quality score >=7/9 | 21 | 2720 | 13.6 | 8.8 | 20.4 | 93.2 |
| Quality score >=8/9 | 15 | 1999 | 14.5 | 8.6 | 23.4 | 93.8 |
| **Sensitivity analyses visual hallucinations in LDB** | | |  |  |  |  |
| **Analyses** | **# studies** | **# participants** | **prevalence (%)** | **LCL 95%** | **ULD 95%** | **I^2^ (%)** |
| 2000 and later studies only | 21 | 2614 | 39.3 | 28.8 | 50.8 | 95.8 |
| 2005 and later studies only | 16 | 1946 | 39.9 | 26.6 | 54.8 | 96.2 |
| 2010 and later studies only | 12 | 1191 | 41 | 21.5 | 63.8 | 96.8 |
| Cross-sectional studies only | 20 | 2528 | 37.1 | 27 | 48.5 | 95.7 |
| Quality score >=7/9 | 21 | 2620 | 38.9 | 28.7 | 50.1 | 95.5 |
| Quality score >=8/9 | 15 | 1899 | 41.7 | 28.8 | 55.7 | 96 |

**Supplementary table 4** Results of sensitivity analyses which demonstrated robustness of our estimates. Including studies only published from 2010 onwards or those of study quality score of 8 or 9/9 produced effect size estimates of around 3% higher for both auditory and visual hallucinations, suggesting our pooled prevalence estimates may be conservative with respect to the true prevalence in the population.
